# Carotenoid Interactions with PCSK9: Exploring Novel Cholesterol-Lowering Strategies

**DOI:** 10.3390/ph17121597

**Published:** 2024-11-27

**Authors:** Alessandro Medoro, Giovanni Scapagnini, Simone Brogi, Tassadaq Hussain Jafar, Truong Tan Trung, Luciano Saso, Sergio Davinelli

**Affiliations:** 1 Department of Medicine and Health Sciences “V. Tiberio”, University of Molise, 86100 Campobasso, Italy; alessandro.medoro@unimol.it (A.M.); giovanni.scapagnini@unimol.it (G.S.); t.jafar@studenti.unimol.it (T.H.J.); 2 Department of Pharmacy, University of Pisa, 56126 Pisa, Italy; simone.brogi@unipi.it; 3 Laboratory of Computation and Nanoscience, Dong Nai Technology University, Bien Hoa City 810000, Vietnam; truongtantrung@dntu.edu.vn; 4 Department of Physiology and Pharmacology “Vittorio Erspamer”, La Sapienza University, 00185 Rome, Italy; luciano.saso@uniroma1.it

**Keywords:** carotenoids, astaxanthin, PCSK9, cholesterol, atherosclerosis

## Abstract

**Background/Objectives**: This study investigated the potential of green algae-derived carotenoids as natural inhibitors of the proprotein convertase subtilisin/kexin type 9 (PCSK9), a key regulator of cholesterol metabolism. PCSK9 promotes the degradation of low-density lipoprotein receptors (LDLR), thereby increasing blood cholesterol levels and elevating the risk of cardiovascular diseases. **Methods**/**Results**: We screened the pharmacophore fit score of 27 carotenoids with PCSK9 and identified 14 that were analyzed for binding affinity and molecular interactions. Astaxanthin, siphonaxanthin, and prasinoxanthin were identified as the top candidates, demonstrating strong binding affinity (−10.5, −10.3, and −9.4 Kcal/mol, respectively) and stable interactions with several known key residues within the active site of PCSK9, including Pro-331, Arg-357, Cys-358, Val-359, Asp-360, Ile-416, Leu-436, Thr-437, Pro-438, Leu-440, Arg-458, Val-460, Trp-461, Arg-476, Cys-477, Ala-478, Ala-649, Val-650, and Asp-651. Density functional theory analysis confirmed the stability of astaxanthin and its favorable electronic properties, suggesting its potential as an effective inhibitor. Molecular dynamics simulations of the PCSK9–astaxanthin complex revealed sustained structural stability and key interactions critical for maintaining the functional integrity of the protein. **Conclusions**: These findings provide evidence that specific carotenoids, particularly astaxanthin, may offer a cost-effective alternative to existing PCSK9 inhibitors, providing a potential approach for managing cholesterol levels and reducing cardiovascular risk. Pre-clinical and clinical validations are required to confirm the therapeutic potential of these compounds.

## 1. Introduction

Protein convertase subtilisin/kexin type 9 (PCSK9) is a serine protease integral to cholesterol metabolism, atherosclerosis, and cardiovascular health. PCSK9 modulates the degradation of low-density lipoprotein receptors (LDLR) on hepatocyte surfaces, thereby influencing the clearance of low-density lipoprotein cholesterol (LDL-C) from the bloodstream [1]. Elevated LDL-C levels are a significant risk factor for atherosclerosis, contributing to cardiovascular diseases that continue to be the leading cause of mortality and morbidity globally [2,3].

Structurally, PCSK9 comprises 692 amino acids, including a 30-residue signal peptide, and contains several functional domains: the prodomain, catalytic domain, and C-terminal domain. These domains play distinct roles in protein activity. The prodomain (residues 31–152) remains closely associated with the catalytic domain, which spans residues 153–451. The C-terminal domain encompasses residues 452–692 [4]. The catalytic domain is particularly critical for the interaction with LDLR, facilitating its degradation and consequently elevating LDL-C levels in the bloodstream. Specific residues within these domains are crucial for PCSK9’s binding and regulatory mechanisms, rendering them prime targets for therapeutic intervention. PCSK9 is synthesized in the endoplasmic reticulum as a zymogen, undergoes autocatalytic cleavage, and remains associated with its prodomain throughout the secretory pathway [5]. As a protease, PCSK9 degrades LDLR, impeding LDL binding and promoting cholesterol accumulation, thereby increasing the risk of coronary artery disease (CAD) [6,7,8,9]. Inhibition of PCSK9 has been validated as an effective strategy for managing cholesterol levels, as evidenced by the efficacy of monoclonal antibodies, such as evolocumab and alirocumab, which target PCSK9. However, these therapies are costly and require subcutaneous administration, prompting the search for alternative, cost-effective, and orally bioavailable inhibitors [10,11,12,13].

Carotenoids and their conversion products modulate adipose tissue biology by reducing adipogenesis, stimulating the emergence of thermogenically active cells in white adipose tissue, and increasing thermogenesis in brown adipose tissue. These compounds exhibit anti-inflammatory and antioxidant properties, improve lipid metabolism, and mitigate obesity-associated metabolic complications, such as insulin resistance and dyslipidemia. Their effects are mediated through enzymatic activity, such as β-carotene-15,15′-dioxygenase (BCO1) and β-carotene-9′,10′-oxygenase (BCO2), and involve the regulation of key pathways, including peroxisome proliferator-activated receptor gamma (PPARγ) and uncoupling protein 1 (UCP1) [14]. Several carotenoids may modulate cholesterol metabolism by targeting PCSK9. For example, lycopene reduces PCSK9 expression via proteasomal degradation of hepatocyte nuclear factor 1α (HNF1α), leading to decreased LDL-C and inflammation, thus supporting cardiovascular health [15,16].

Carotenoids, particularly those derived from seaweed, are being investigated as potential natural inhibitors of PCSK9, offering a novel approach to cholesterol management [15]. Although the direct effects of specific carotenoids, such as astaxanthin, on PCSK9 and cholesterol levels have not been extensively documented, preliminary studies suggest that carotenoids may modulate lipid metabolism and reduce oxidative stress—an important factor in the development of atherosclerosis [17,18]. Their antioxidant properties may provide additional cardiovascular protection, enhancing their potential as a non-pharmacological strategy [19]. While research specifically linking carotenoids to PCSK9 inhibition is in its early stages, emerging evidence suggests that certain carotenoids may influence cholesterol metabolism through various mechanisms, including transport and storage. Indeed, carotenoids are transported in plasma by lipoproteins, such as chylomicrons, LDL, very low-density lipoproteins (VLDL), and high-density lipoproteins (HDL). Additionally, a recent study demonstrated that LDLR is a crucial regulator of carotenoid homeostasis, including the storage of carotenoids in the liver and adipose tissue, as well as their elimination via feces. Therefore, the involvement of LDLR in the uptake and distribution of carotenoids suggests that these bioactive molecules may interact directly with pathways critical for cholesterol metabolism [20]. Although dependent on sex, carotenoids, through their conversion to retinoic acid (RA), influence cholesterol metabolism by modulating the expression of key genes and receptors, including ATP-binding cassette transporter subfamily A member 1 (ABCA1), subfamily G member 1 (ABCG1), cluster of differentiation 36 (CD36), and scavenger receptor class B type 1 (SCARB1). ABCA1 and ABCG1 are involved in cholesterol efflux, with ABCA1 contributing to HDL formation and ABCG1 playing a role in macrophage cholesterol metabolism. CD36 facilitates oxidized LDL uptake, while SCARB1 regulates selective HDL cholesterol uptake, influencing overall cholesterol balance and cardiovascular health [21].

Recent studies have demonstrated that microalgae carotenoids may improve the activity of antioxidant enzymes, such as glutathione peroxidase (GPx) and glutathione reductase (GR), and modulate cholesterol metabolism by lowering lipid peroxidation, thereby providing beneficial effects against oxidative stress and dyslipidemia [22]. Astaxanthin, an antioxidant carotenoid found in marine organisms, such as microalgae (e.g., *Haematococcus pluvialis*) and seafood, has demonstrated promise in modulating lipid profiles in animal models. Studies have indicated that astaxanthin can lower LDL-C and increase cholesterol, suggesting a beneficial shift in lipid metabolism [17,19]. Furthermore, astaxanthin reduces oxidative stress and inflammation, which are critical contributors to atherosclerosis and other cardiovascular diseases. By mitigating oxidative stress, carotenoids may prevent the oxidation of LDL particles, a key event in the initiation of atherosclerotic plaques, thereby indirectly affecting PCSK9 levels and activity [23].

The exploration of carotenoids in this context represents a promising avenue of research. Therefore, this study aims to investigate, through in silico techniques, the potential of carotenoids from green algae to inhibit the enzymatic activity of PCSK9. This study proposes a natural, dietary-based approach to cholesterol reduction, potentially offering a cost-effective and non-invasive alternative to existing treatments.

## 2. Results and Discussion

### 2.1. Selection of Green Algae-Derived Carotenoid Potential Inhibitors of PCSK9

Fourteen green algae-derived carotenoids (adonirubin, adonixanthin, antheraxanthin, astaxanthin, echinenone, lutein, neoxanthin, nostoxanthin, peridinin, peridinol, prasinoxanthin, pyrrhoxanthin, siphonaxanthin, and zeaxanthin) were selected from the initial 27 carotenoids based on their ligand-based pharmacophore fit scores (Supplementary Table S1).

### 2.2. Molecular Docking Analysis

Molecular docking analyses of fourteen marine-derived green algae compounds were conducted using AutoDock Vina. The docked complexes were evaluated based on their binding affinity, binding energy, hydrogen bonding, and hydrophobic interactions, leading to the selection of the top three compounds for further investigation. The molecular docking studies indicated that all fourteen compounds interacted with PCSK9 active sites and exhibited similar binding regions. Table 1 presents the binding values of all compounds, highlighting those with favorable binding energies. The top three docked complexes were identified as astaxanthin (−10.5 kcal/mol), siphonaxanthin (−10.3 kcal/mol), and prasinoxanthin (−9.4 kcal/mol).

The docking scores and molecular interactions of the three top-ranked compounds with PCSK9 are summarized in Table 2. By evaluating the docking energies, we can predict the optimal conformational positions of these compounds within the active region of PCSK9. The three complexes with the lowest binding scores (astaxanthin, siphonaxanthin, and prasinoxanthin) were further analyzed based on their energy values and molecular bonding interactions, including hydrogen, hydrophobic, and electrostatic interactions. Lower binding energies indicate higher binding affinity and more stable molecular interactions.

**Figure 1:**
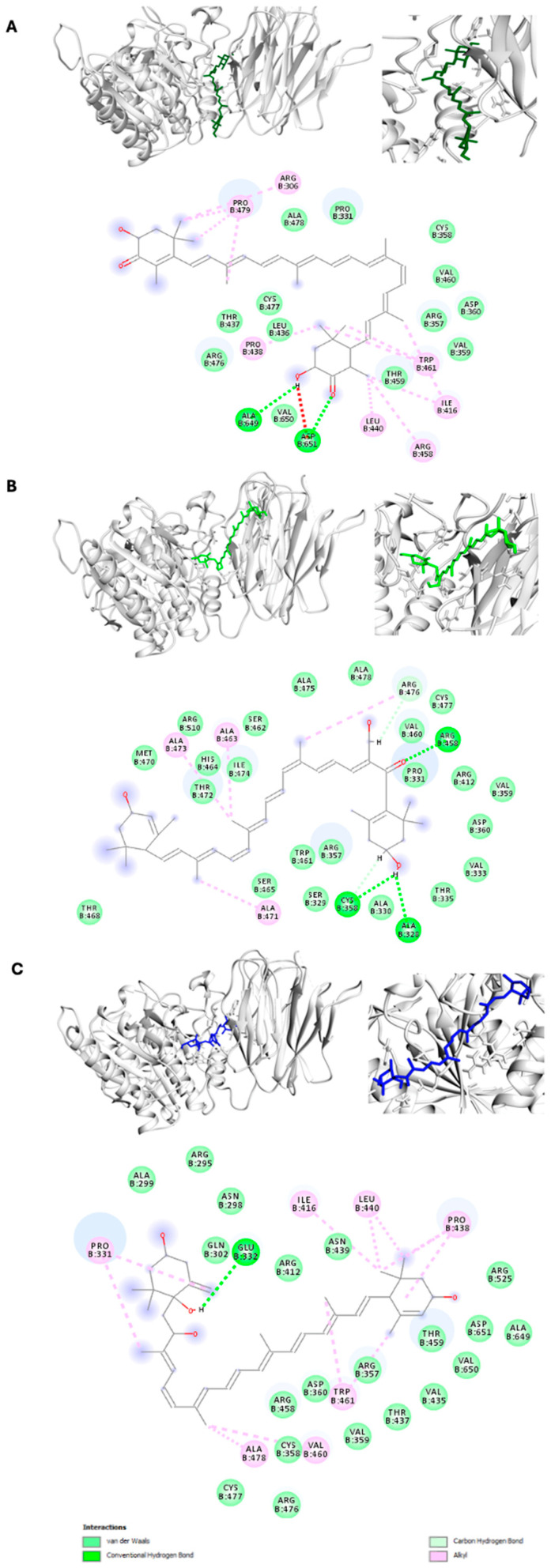
3D and 2D molecular interaction analyses of PCSK9 (grey) with (**A**) astaxanthin (dark green), (**B**) siphonaxanthin (light green), and (**C**) prasinoxanthin (blue). All three compounds showed similar residue interaction with PCSK9 due to the high structural similarity. Astaxanthin and siphonaxanthin showed almost two hydrogen bonds with key residues.

The docking complex of astaxanthin with PCSK9 demonstrated that the compound could effectively bind to the active site of the target protein, exhibiting strong hydrogen bonding and hydrophobic interactions. The binding interactions indicated significant binding energies within the docking complex. Key molecular interactions were identified at the binding site, including residues Arg-306, Pro-331, Arg-357, Cys-358, Val-359, Asp-360, Ile-416, Leu-436, Thr-437, Pro-438, Leu-440, Arg-458, Thr-459, Val-460, Trp-461, Arg-476, Cys-477, Ala-478, Pro-479, Ala-649, Val-650, and Asp-651, as illustrated in Figure 1A. Several critical residues (Arg-306, Pro-331, Arg-357, Cys-358, Val-359, Asp-360, and Ile-416) were identified within the binding region of the catalytic domain of PCSK9. Furthermore, the docking analysis revealed additional molecular interactions involving residues Arg-458, Thr-459, Val-460, Trp-461, Arg-476, Cys-477, Ala-478, and Pro-479, which exhibited strong interactions within the active pocket of the C-terminal binding region of PCSK9. Our findings indicate that astaxanthin showed almost all binding residues have been previously reported as key residues in the inhibition of PCSK9 (6U3X, 6U2N, 6U2P, and 6U38) with cholesterol-lowering agents: Pro-331, Arg-357, Cys-358, Val-359, Asp-360, Ile-416, Leu-436, Thr-437, Pro-438, Leu-440, Arg-458, Val-460, Trp-461, Arg-476, Cys-477, Ala-478, Ala-649, Val-650, and Asp-651 [6,7,8,9,24]. In addition, in the astaxanthin–PCSK9 docking complex, two hydrogen bonds with favorable bond lengths were identified at Ala-649 and Asp-651. Notably, these hydrogen-bonding interactions (Ala-649 and Asp-651) have been reported in the literature [9].

Similarly, siphonaxanthin exhibited favorable hydrogen and hydrophobic interactions with PCSK9, maintaining appropriate distances. Key molecular interactions in the docked complex included residues Ala-328, Ser-329, Ala-330, Pro-331, Val-333, Thr-335, Arg-357, Cys-358, Val-359, Asp-360, Arg-412, Arg-458, Val-460, Trp-461, Ser-462, Ala-463, His-464, Ser-465, Thr-468, Met-470, Ala-471, Thr-472, Ala-473, Ile-474, Ala-475, Arg-476, Cys-477, Ala-478, and Arg-510, as depicted in Figure 1B. Val-359, Asp-360, and Arg-412 were key residues identified in the catalytic binding pockets of PCSK9.

The binding interactions between prasinoxanthin and PCSK9 revealed strong hydrogen bonds and hydrophobic interactions within the docking complex. The overall binding residues identified in this complex included Arg-295, Asn-298, Ala-299, Gln-302, Pro-331, Glu-332, Arg-357, Cys-358, Val-359, Asp-360, Arg-412, Ile-416, Val-435, Thr-437, Pro-438, Asn-439, Leu-440, Arg-458, Thr-459, Val-460, Trp-461, Arg-476, Cys-477, Ala-478, Arg-525, Ala-649, Val-650, and Asp-651, as shown in Figure 1C.

### 2.3. DFT-Based Optimized Structures, Frontier Molecular Orbitals, and Molecular Electrostatic Potential Analysis of Astaxanthin

**Figure 2:**
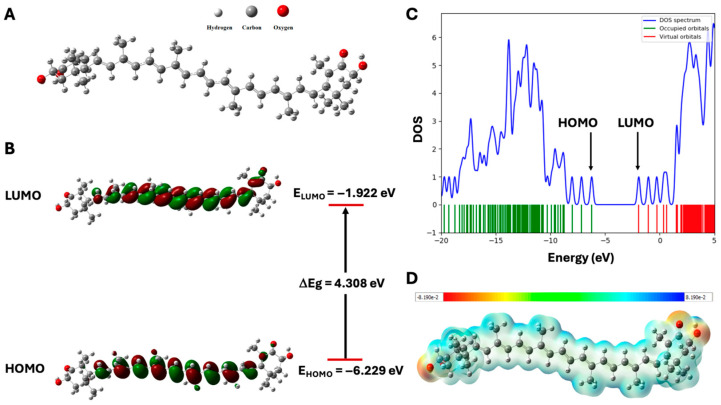
(**A**) Optimized geometry (**B**) HOMO, LUMO orbitals and their energy gap (ΔEg), (**C**) DOS plot diagram, and (**D**) MEP map of astaxanthin by DFT level of calculations.

According to the docking results, astaxanthin appears to be the most promising carotenoids for further in silico analyses. The numbering scheme for the atoms and the optimized geometry of astaxanthin obtained from DFT calculations are presented in Figure 2A. The optimized structures of astaxanthin exhibit non-planar geometries with C1 point group symmetry, which corresponds to a high dipole moment of 2.526 Debye. Figure 2B,C show the HOMO and LUMO molecular surfaces, as well as a density of states (DOS) diagram for the astaxanthin compound.

Table 3 summarizes the calculated values of E_HOMO_, E_LUMO,_ ΔEg, and other global quantum descriptors. Astaxanthin has an energy gap (ΔEg) of 4.308 eV, with HOMO and LUMO energies of −6.229 eV and −1.922 eV, respectively. This substantial energy gap indicates that the compound is stable. The ionization potential (IP) and electron affinity (EA) of astaxanthin were measured at 4.308 eV and 1.922 eV, respectively. Additionally, the chemical hardness (η), chemical potential (μ), chemical softness (S), and electrophilicity index (ω) are calculated to be 2.154, −4.075, 0.232, and 3.856 eV, respectively. These values suggest that the compound is both hard and stable.

The MEP of astaxanthin was also derived from the DFT calculations, with an isovalue electron density of 0.004 atomic units, as shown in Figure 2D. In the MEP surface, the maximum negative region, indicated in red, represents areas prone to electrophilic attack; whereas the maximum positive region, shown in blue, corresponds to nucleophilic attack. The energy range from the maximum negative to the maximum positive potential spans −8.190 × 10^−2^ to 8.190 × 10^−2^ a.u., highlighting the nucleophilic regions associated with the oxygen atoms (O19, O20, O43, and O44). A more negative electrostatic potential promotes nucleophilic attraction.

To investigate the relationships among molecular structures and non-linear optic properties (NLO), the total polarizability (α_tot_), anisotropy of the polarizability (Δα), and first-order hyperpolarizability (β_tot_) were calculated from the Gaussian output. The polarizability and first-order hyperpolarizability values calculated by DFT/M052X/6-311++G(d,p) are presented in Table 4. Atomic units (a.u) were converted into electrostatic units (esu) as (for α, 1 a.u. = 0.1482 × 10^−24^ esu; for β, 1 a.u. = 0.008639 × 10^−30^ esu). The calculated total polarizability (α_tot_) and anisotropy of the polarizability (Δα) are 17.958 × 10^−24^ esu and 27.746 × 10^−24^ esu. The calculated value of first-order hyperpolarizability (β_tot_) is 0.962 × 10^−30^ esu, which is five times greater than that of urea (0.1947 × 10^−30^ esu). Overall, these results confirm that astaxanthin has good NLO properties.

### 2.4. Molecular Dynamics Simulation of PCSK9–Astaxanthin Docking Complex

**Figure 3:**
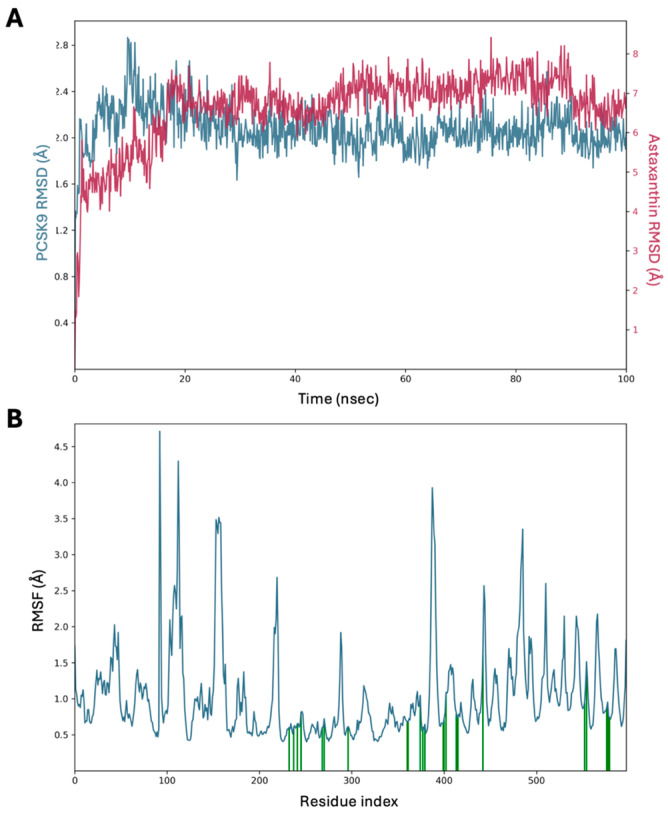
(**A**) RMSD analysis of simulation complex PCSK9–astaxanthin. The left Y-axis showed the variation of PCSK9 RMSD through time, while the right Y-axis showed the variation of astaxanthin RMSD through time. (**B**) The analysis of RMSF during simulation complex of PCSK9–astaxanthin.

Monitoring the RMSD of a protein offers valuable insights into its structural conformation throughout a simulation. RMSD analysis can help determine whether the simulation has reached equilibrium, as fluctuations stabilize around a thermal average structure by the end of the simulation. For small globular proteins, deviations of 1–3 Å are generally acceptable; however, larger shifts may indicate significant conformational changes. In Figure 3A, the RMSD values indicate the average deviation of the astaxanthin–PCSK9 simulation complex. The astaxanthin–PCSK9 complex exhibited structural stability throughout the simulation, with small fluctuations. The RMSD values (2.4 ± 0.4 Å) suggest that PCSK9 underwent minor local conformational changes while maintaining its overall folding and binding stability. Notably, between 70 ns and 80 ns of the simulation, the astaxanthin complex with PCSK9 exhibited the highest RMSD value (2.6 ± 0.4 Å), indicating small conformational changes. By contrast, PCSK9 displayed greater variation in protein conformation, as evidenced by its higher RMSD value (2.8 ± 0.5 Å), suggesting conformational changes within the overall docked complex.

RMSF analysis is particularly useful for characterizing local changes along the protein chain. In the RMSF plots, the peaks represent regions of protein that exhibit the greatest fluctuations during the simulation. Typically, the N- and C-terminal regions exhibit greater fluctuations than other parts of the protein. Secondary structural elements, such as alpha helices and beta strands, tend to be more rigid than unstructured regions, resulting in less fluctuation than loop regions. Figure 3B presents the RMSF plot for PCSK9, highlighting the regions with the highest flexibility, particularly at the N- and C-terminal ends. The green vertical bars in the ligand contact section indicate the protein residues that interact with the ligand, whereas the RMSF provides detailed fluctuation data for each ligand atom. Notably, the astaxanthin–PCSK9 complex exhibited RMSF values ≤ 2.5 Å, indicating a firmly bound ligand with binding stability and rigidity in the PCSK9 structure. The interactive residues between residues 250–300 and 375–425 exhibited flexibility below 1.0 Å, while higher fluctuations (4.0–4.5 Å) were observed between residues 100–120.

**Figure 4:**
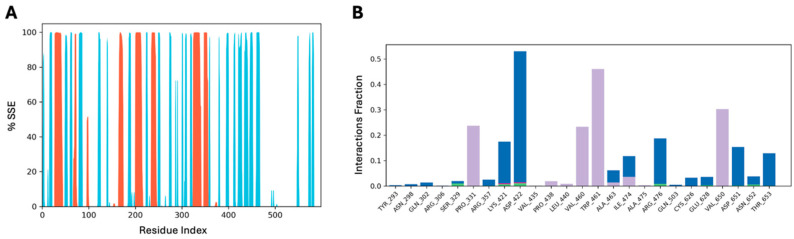
(**A**) PCSK9 SSE (%) distribution by residue index throughout the simulation complex. Red columns indicate the alpha helices, and light blue columns indicate the beta strands. (**B**) PCSK9–astaxanthin contact histogram. Hydrogen bonds are in green, hydrophobic interaction in purple, and water bridges in blue.

The secondary structure elements (SSE) analysis, including alpha helices and beta strands throughout the simulation, is displayed in Figure 4. The SSE distribution is presented as the residue index across the protein structure, with bars representing the SSE composition of each residue. The SSE of PCSK9 indicates stable structural confirmation, with overall trajectories showing 14.33% alpha helices (orange) and 21.66% beta strands (blue), along with loop regions represented as white spaces. Figure 4 highlights key protein–ligand interactions within the complex, focusing on interactions occurring for >20% of the simulation time. These protein–ligand interactions, or “contacts”, are categorized into four types: hydrogen bonds, hydrophobic interactions, ionic interactions, and water bridges. The stacked bar charts are normalized to the trajectory, where a value of 0.5 indicates that a specific interaction is maintained for 50% of the simulation time, as observed for Asp-422. The analysis revealed key residual interactions, including hydrogen bonding, and hydrophobic, ionic, and water bridge interactions in the astaxanthin–PCSK9 complex. The protein–ligand contact histogram illustrates important residual interactions, with hydrogen bonds shown in green, hydrophobic interactions in purple, ionic interactions in deep pink, and water bridges in blue. The green bars indicate that residues Ser-329, Lys-421, Asp-422, Arg-476, Cys-628, Asp-651, and Asn-652 maintained a minimum distance of under 2.5 Å. Key hydrophobic residues (Pro-331, Val-460, Trp-461, and Val-650) were also analyzed in the complex and played a crucial role in the binding stability of astaxanthin–PCSK9 molecular interactions. The current simulation identified key binding residues within the binding domain of PCSK9, including Tyr-293, Asn-298, Gln-302, Arg-306, Ser-329, Pro-331, Arg-357, Lys-421, Asp-422, Val-435, Pro-438, Leu-440, Val-460, Trp-461, Ala-463, Ile-474, Ala-475, Arg-476, Gln-503, Cys-626, Glu-628, Val-650, Asp-651, Asn-652, and Thr-653. These residues play significant roles in the binding and degradation of LDLR and contribute to the stability of the simulation complex, binding flexibility, and binding confirmation. The astaxanthin–PCSK9 simulation complex underscores the importance of these residues in maintaining the functional integrity of PCSK9 and its domains. In the context of the astaxanthin–PCSK9 complex, residues Asn-298, Gln-302, Arg-306, Ser-329, Pro-331, Arg-357, Lys-421, and Asp-422 are critical for binding in the catalytic domain as indicated by molecular docking studies. Additionally, residues Val-435, Pro-438, Leu-440, Val-460, Trp-461, Ala-463, Ile-474, Ala-475, Arg-476, Gln-503, Cys-626, Glu-628, Val-650, Asp-651, Asn-652, and Thr-653 contribute to C-terminal interactions that stabilize the PCSK9–LDLR complex. These binding interactions consistently identified these residues as hotspots for ligand binding, highlighting their potential as targets for drug discovery. Furthermore, the C-terminal domain of PCSK9 (the cysteine-rich domain) is crucial for ligand binding because it plays a vital role in maintaining the structural and functional integrity of the protein through these interactive residues.

**Figure 5:**
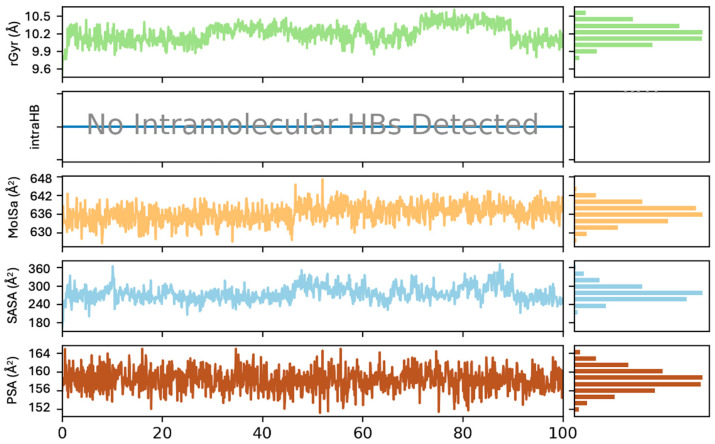
Graphical representations of rGyr, IntraHB, MolSA, SASA, and PSA.

The potential activity of astaxanthin was assessed using the RMSD, rGyr, MolSA, SASA, and PSA values in the astaxanthin–PCSK9 complex from 0 to 100 ns (Figure 5). These analyses provide valuable insights into the stability and binding of astaxanthin to PCSK9 in the simulation system. In the graphical representations, values are displayed on the left side, histogram distributions are displayed on the right side, and the simulation time at the bottom corresponds to different peaks in the data.

In Figure 5, the green bars representing rGyr indicate the compactness of the ligand throughout the simulation, suggesting a stable equilibrium between the molecular surface area and binding modes. Initially, the rGyr values indicate minor fluctuations between 9.6 Å and 9.9 Å. After 60 ns, significant fluctuations up to 10.5 Å suggest that the ligand unfolds or continuously changes its conformation to maintain stability and flexibility within the active site. Stable fluctuations around 10.2 Å indicate a compact structure throughout the simulation. The histogram distribution of rGyr values around 9.9 Å to 10.2 Å confirms the maintained compactness of the ligand. The stable rGyr values suggest that the ligand underwent significant conformational changes to support binding interactions.

MolSA analysis was used to assess the ligand size and shape during the simulation. The MolSA values show minor fluctuations due to conformational changes. During the first 50 ns, the MolSA values remain stable around 630 Å^2^ to 636 Å^2^, indicating consistent molecular interactions with the protein and solvent. Between 45 and 50 ns, significant fluctuations between 642 Å^2^ and 648 Å^2^ are observed, likely due to structural changes. After 60 ns, the MolSA values stabilize between 636 Å^2^ and 640 Å^2^. The histogram distribution further reflects a stable surface area of around 636 Å^2^.

SASA measures the ligand’s surface area exposed to the solvent within the protein’s active pocket. The SASA values ranged from 180 Å^2^ to 360 Å^2^, which is generally acceptable. In the first 50 ns, SASA values indicate a lower surface area (180 Å^2^ to 300 Å^2^), suggesting that the ligand is more buried within the protein active site. High fluctuations above 300 Å^2^ between 40 ns and 60 ns suggest minor adjustments in the ligand’s conformation, indicating increased exposure to the solvent. The histogram distribution shows stable ligand interaction with the solvent around 250 Å^2^ to 300 Å^2^ throughout the simulation, maintaining a consistent orientation within the PCSK9 binding pocket.

PSA was used to assess the ligand’s polar regions and their interactions with the solvent within the active binding site. Throughout most of the simulation, PSA values between 152 Å^2^ and 160 Å^2^ indicate a stable surface area around the solvent, which is crucial for solubility and ligand binding. According to the PSA graph, these values suggest a balanced ligand polarity toward the protein. In some phases, higher polarity values (up to 164 Å^2^) suggest increased exposure to the solvent, which could affect binding affinity. However, at the end of the simulation, the ligand maintains balanced polar interactions. The rGyr and MolSA graphs indicate possible binding interactions, whereas the SASA and PSA graphs suggest a stable conformational position within the PCSK9 binding pocket under limited solvent exposure. Notably, no internal hydrogen bonds (HBS) were detected between astaxanthin and PCSK9 during the simulation. The absence of intermolecular hydrogen bonds suggests that the ligand’s binding conformation, flexibility, and stability are primarily driven by other molecular interactions, including external hydrogen bonds, hydrophobic interactions, and water bridges with the protein. Overall, the stability of astaxanthin within the PCSK9 active site indicates its potential to strongly bind to the protein.

## 3. Materials and Methods

### 3.1. Retrieval of the 3D Structure of PCSK9

To ensure the accuracy and relevance of the structural analysis, all available 3D structures of PCSK9 were thoroughly reviewed from the Protein Data Bank (PDB), focusing specifically on those with defined binding sites. After evaluating various structural models, the PDB ID 6U26 (https://www.rcsb.org/structure/6u26, accessed on 19 May 2024) was selected as the most appropriate model for further studies. This structure was selected based on its resolution (1.59 Å), which ensures precise visualization of the protein–ligand interactions, and its well-defined binding pockets. The active site of the protein is where the interaction between PCSK9 and the EGF-A domain of LDLR occurs [24]. After retrieving the 3D structure of PCSK9, we validated its reliability using computational methods. The structure was prepared using UCSF Chimera v1.12, during which we analyzed it for missing residues and performed refinement. First, water molecules, additional chains, and extraneous ligands present in the crystal structure were removed to eliminate interference with docking calculations. Following this, hydrogen atoms were added to the protein structures to account for correct protonation states, for both polar and non-polar residues. Next, atomic charges were assigned through the AMBER force field (AMBER ff14SB) using the AM1-BCC (Austin Model 1—Bond Charge Correction) method, which applies partial charges with bond charge corrections, enhancing the accuracy of the molecular modeling. Additionally, the 3D structure underwent energy minimization in UCSF Chimera v1.12, utilizing the 1000 steepest descent and 1000 conjugate gradient steps with Amber force field parameters [25].

### 3.2. Compound Selection, Structure Preparation, and Pharmacophore Fit Score-Based Screening

Twenty-seven carotenoids derived from green algae were selected based on a review of the literature and the CHEMnetBASE—Dictionary of Marine Natural Products. The selected carotenoids include: adonirubin, adonixanthin, alloxanthin, antheraxanthin, astaxanthin, β-cryptoxanthin, caloxanthin, canthaxanthin, crocoxanthin, diadinoxanthin, diatoxanthin, echinenone, fucoxanthin, loroxanthin, lutein, monadoxanthin, neoxanthin, nostoxanthin, peridinin, peridinol, prasinoxanthin, pyrrhoxanthin, siphonaxanthin, vaucheriaxanthin, violaxanthin, zeaxanthin, and zeinoxanthin. To identify potential PCSK9 inhibitors among these carotenoids, we assessed the ligand-based pharmacophore fit score using LigandScout v.4.4. This score is a reliable and efficient method for evaluating the potential of a compound to bind to a protein’s active site. A higher fit score indicates better geometric alignment of the compound, which correlates with an accurate prediction of the molecular docking score [26,27].

We retrieved the 2D structures of the 13 carotenoids with the highest pharmacophore fit scores from the PubChem database to obtain structural information. Additionally, all compounds were sketched using ChemDraw ultra v12.0 and exported in PDB format. The refinement and geometry optimization of these compounds were confirmed using Chem3D Pro v12.0 and UCSF Chimera v1.12, respectively. Energy minimization was conducted over 1500 runs, employing the steepest descent and conjugate gradient methods for all compounds [18].

### 3.3. Molecular Docking Analyses

Molecular docking studies were conducted using the AutoDock Vina tool v4.2. The complete process was performed in five steps according to the NAMD protocols. The grid box for the docking simulations was configured to a size of 75 × 75 × 75 Å along the X = 33.87, Y = 23.67, and Z = 25.32 axes, to adequately cover the known binding pocket of PCSK9 [24]. A grid spacing value of 0.650 Å was applied to precisely define the docking environment. For each docking experiment, a total of 100 docking runs were performed, and polar hydrogen atoms were added to the PCSK9 protein. Default parameters were utilized, with the genetic algorithm serving as the primary search protocol. The resulting docked complexes were analyzed based on binding affinities and energy values, as well as the interactive behaviors of ligands, including hydrogen, and hydrophobic interactions. Additionally, the binding conformations of all selected carotenoids within the PCSK9 binding site were examined, leading to the identification of three carotenoids—astaxanthin, siphonaxanthin, and prasinoxanthin—as the top hits based on their binding scores. The graphical representations of the docked complexes were visualized using UCSF Chimera v1.12, Discovery Studio v2022, and LigPlot v2.6 [28,29].

### 3.4. Density Functional Theory

All density functional theory (DFT) computations for this study were performed using the Gaussian 16 (revision C.01) software package [30]. Ground state geometries were determined in the gas phase using the DFT method with the M052X local density functional and the 6-311++G(d,p) basis set [31]. Natural bond orbital (NBO) analysis was conducted using NBO 5.1, which is integrated into the Gaussian software, to examine the frontier molecular orbitals at the M052X/def2TZVPP level of theory. The energy values of the highest occupied molecular orbital (HOMO) and the lowest unoccupied molecular orbital (LUMO) were utilized to calculate various quantum chemical properties, including ionization potential (IP), electron affinity (EA), energy gap (ΔEg), electronegativity (χ), chemical hardness (η), chemical potential (μ), chemical softness (S), and electrophilicity index (ω), as detailed in previous studies [32]. Additionally, the molecular electrostatic potential (MEP) was calculated at the same level of theory. Density of states (DOS) plots were generated using GaussSum v3.0, and the results were visualized using GaussView v6.0 [27].

### 3.5. Molecular Dynamics Simulation

Molecular dynamics simulations were conducted for a standard duration of 100 ns using Desmond (Schrödinger LLC, New York, NY, USA). These simulations compute atomic movements over time by integrating Newton’s classical equations of motion [33]. The top-docked complex, astaxanthin–PCSK9, was prepared using the Protein Preparation Wizard in Maestro, which facilitated complex optimization and minimization. All simulation systems were constructed using the system builder tool. The Transferable Intermolecular Interaction Potential 3 Points (TIP3P) solvent model, housed in an orthorhombic box, was selected for the system. The OPLS 2005 force field was used in the simulation [34]. To ensure the model’s neutrality and to replicate physiological conditions, 0.15 M sodium chloride (NaCl) was added as counter ions. The NPT ensemble was utilized, maintaining a temperature of 300 K and a pressure of 1 atm throughout the simulation. The model was relaxed before the simulation, and the trajectories were saved for subsequent analysis. The stability of the simulation was assessed by comparing the root mean square deviation (RMSD) of the protein and ligand over time. Molecular dynamics of 100 ns duration were run with additional analyses of complex including secondary structure elements (SSE), protein–ligand contact analysis, and detailed analyses of ligand properties: root mean square fluctuation (RMSF), radius of gyration (rGyr), intramolecular hydrogen bonds, molecular surface area (MolSA), solvent accessible surface area (SASA), and polar surface area (PSA).

## 4. Conclusions

In summary, this study investigated the potential of carotenoids derived from green algae, specifically astaxanthin, siphonaxanthin, and prasinoxanthin, as natural inhibitors of PCSK9. Through in silico molecular docking, binding affinity analyses, and molecular dynamics simulations, these compounds demonstrated promising interactions with key residues in the active sites of PCSK9. Among the tested compounds, astaxanthin, a naturally occurring carotenoid found in marine organisms such as algae and seafood, showed the most favorable binding interactions, stability, and molecular properties, suggesting it as a viable candidate for further exploration in cholesterol management.

Its ability to interact with PCSK9 and potentially inhibit its function suggests that it could serve as a complementary approach to traditional cholesterol-lowering therapies. Unlike monoclonal antibodies currently used to inhibit PCSK9—which are costly and require injections—astaxanthin can be introduced through the diet, making it a more accessible and non-invasive option. The antioxidant properties of astaxanthin add an additional layer of cardiovascular protection, as it helps reduce oxidative stress and inflammation, both critical factors in the progression of atherosclerosis. This dual action—modulating cholesterol metabolism and providing antioxidant support—positions astaxanthin as a valuable candidate for non-pharmacological strategies in managing cholesterol levels and reducing cardiovascular risk.

However, this study presents certain limitations. The in-silico approach, while valuable for initial screening and hypothesis generation, does not replicate the full complexity of an in vivo environment. Molecular interactions, absorption, metabolism, and potential side effects cannot be fully accounted for in a computational model. Future studies should include in vitro and in vivo analyses to validate these findings and assess their efficacy in cholesterol management.

## Figures and Tables

**Table 1:** Calculation of the binding affinities of selected green algae carotenoids against PCSK9 utilizing AutoDock Vina v4.2.

	Carotenoids	Binding Affinities (Kcal/mol)
1	Astaxanthin	−10.5
2	Siphonaxanthin	−10.3
3	Prasinoxanthin	−9.4
4	Zeaxanthin	−9.1
5	Nostoxanthin	−8.8
6	Neoxanthin	−8.6
7	Peridinol	−8.6
8	Pyrrhoxanthin	−8.5
9	Peridinin	−8.3
10	Antheraxanthin	−8.2
11	Adonixanthin	−8.0
12	Lutein	−7.6
13	Echinenone	−7.5
14	Adonirubin	−7.1

**Table 2:** Analyses of the binding residues in docked complexes of targeted carotenoids with PCSK9. Bold residues indicate potential hydrogen bonds.

Carotenoids	Binding Residues in Docking Complexes
Astaxanthin	Arg-306, Pro-331, Arg-357, Cys-358, Val-359, Asp-360, Ile-416, Leu-436, Thr-437, Pro-438, Leu-440, Arg-458, Thr-459, Val-460, Trp-461, Arg-476, Cys-477, Ala-478, Pro-479, **Ala-649**, Val-650, **Asp-651**
Siphonaxanthin	**Ala-328**, Ser-329, Ala-330, Pro-331, Val-333, Thr-335, Arg-357, **Cys-358**, Val-359, Asp-360, Arg-412, **Arg-458**, Val-460, Trp-461, Ser-462, Ala-463, His-464, Ser-465, Thr-468, Met-470, Ala-471, Thr-472, Ala-473, Ile-474, Ala-475, Arg-476, Cys-477, Ala-478, Arg-510
Prasinoxanthin	Arg-295, Asn-298, Ala-299, Gln-302, Pro-331, **Glu-332**, Arg-357, Cys-358, Val-359, Asp-360, Arg-412, Ile-416, Val-435, Thr-437, Pro-438, Asn-439, Leu-440, Arg-458, Thr-459, Val-460, Trp-461, Arg-476, Cys-477, Ala-478, Arg-525, Ala-649, Val-650, Asp-651

**Table 3:** Quantum chemical parameters of astaxanthin.

Compound Properties	Quantum Description	M052X/def2TZVPP
E_HOMO_ (eV)	Energy of HOMO	−6.229
E_LUMO_ (eV)	Energy of LUMO	−1.922
ΔEg—Energy gap (eV)	ΔEg = E_LUMO_ − E_HOMO_	4.308
IP—Ionization potential (eV)	IP = −E_HOMO_	6.229
EA—Electron affinity (eV)	EA = −E_LUMO_	1.922
χ—Electronegativity (eV)	χ = −(E_LUMO_ + E_HOMO_)/2	4.075
η—Chemical hardness (eV)	η = (E_LUMO_ − E_HOMO_)/2	2.154
μ—Chemical potential (eV)	μ = −χ = (E_LUMO_ + E_HOMO_)/2	−4.075
S—Chemical softness (eV)	S = 1/2η	0.232
ω—Electrophilicity index (eV)	ω = μ2/2η	3.856

**Table 4:** Calculated polarizability (α) and first-order hyperpolarizability (β) of astaxanthin.

Polarizability (α)			First Order Hyperpolarizability (β)		
Parameter	a.u.	esu (×10^−24^)	Parameter	a.u.	esu (×10^−30^)
αxx	1457.750	216.016	βxxx	13,071.100	112.924
αxy	−316.677	−46.927	βxxy	−2298.600	−19.858
αyy	474.804	70.359	βxyy	513.543	4.437
αxz	−348.121	−51.586	βyyy	−104.064	−0.899
αyz	53.503	7.928	βxxz	−585.550	−50.587
αzz	520.590	77.144	βxyz	896.348	7.744
αtot	817.715	17.958	βyyz	−144.524	−1.249
Δα	1263.440	27.746	βxzz	2749.080	23.750
			βyzz	−355.390	−3.070
			βzzz	−1217.920	−10.522
			βtot	11,377.741	0.962
			β0	6826.645	0.005

## Data Availability

Data generated during the current study are available from the corresponding author on reasonable request.

## Supplementary Materials

The following supporting information can be downloaded at: https://www.mdpi.com/article/10.3390/ph17121597/s1, Table S1: Calculation of pharmacophore fit score by LigandScout software.
