# Supplementary material for: Carotenoid Interactions with PCSK9: Exploring Novel Cholesterol-Lowering Strategies
Source: Pharmaceuticals (Basel). 2024 Nov 27;17(12):1597. doi: 10.3390/ph17121597 (PMC11676125; doi:10.3390/ph17121597)
Supplement: Supplementary file 1 [file pharmaceuticals-17-01597-s001.zip › pharmaceuticals-3309680-supplementary.pdf]

Supplementary materials

Table S1. Calculation of pharmacophore fit score by LigandScout software.

	**Carotenoids**	**Pharmacophore fit score**
1	Adonirubin	67.88
2	Adonixanthin	67.60
3	Alloxanthin	67.32
4	Antheraxanthin	67.71
5	Astaxanthin	67.71
6	β-Cryptoxanthin	66.01
7	Caloxanthin	67.14
8	Canthaxanthin	No score identify
9	Crocoxanthin	65.86
10	Diadinoxanthin	67.32
11	Diatoxanthin	67.37
12	Echinenone	67.70
13	Fucoxanthin	67.31
14	Loroxanthin	67.39
15	Lutein	67.61
16	Monadoxanthin	67.39
17	Neoxanthin	67.69
18	Nostoxanthin	67.70
19	Peridinin	67.69
20	Peridinol	67.70
21	Prasinoxanthin	67.63
22	Pyrrhoxanthin	67.62
23	Vaucheriaxanthin	67.32
24	Violaxanthin	67.31
25	Zeaxanthin	67.70
26	Zeinoxanthin	67.40
27	Siphonaxanthin	67.69
